# Sex differences in the impact of controlling nutritional status score on diabetic retinopathy: findings of 2003–2018 National Health and Nutrition Examination Survey

**DOI:** 10.3389/fnut.2025.1597521

**Published:** 2025-05-16

**Authors:** Yu-Nan Han, Shui-Ping Li, Yong-Xiang Wang, Zui-Xuan Xiao, Lin Li

**Affiliations:** ^1^Department of Endocrinology, The First Affiliated Hospital of Yangtze University, Jingzhou, Hubei, China; ^2^Department of Medicine, Yangtze University, Jingzhou, Hubei, China

**Keywords:** sex differences, CONUT score, diabetes, diabetic retinopathy, NHANES

## Abstract

**Background:**

Nutritional status plays a crucial role in the progression of diabetes complications. This study assessed sex differences in Controlled Nutritional Status (CONUT) score and diabetic retinopathy (DR).

**Methods:**

Clinical data between 2003 and 2018 were retrieved from the National Health Nutritional Examination Survey (NHANES) database. The association of CONUT score with DR was analyzed by multivariate weighted logistic regression with restricted cubic splines (RCS). The impact of CONUT scores on DR outcomes in male and female patients was evaluated by subgroup analyses and interaction tests.

**Results:**

A total of 3,762 participants were included in this study. After adjusting for all covariates, a higher CONUT score was positively associated with DR risk in women (OR = 1.88, 95% CI: 1.13–3.15), while no significant association between CONUT score and DR was observed in male participants and the overall participant population. In addition, RCS regression showed a linear positive correlation between CONUT score and DR risk in women (*P*-Nonlinear: 0.722). Subgroup analyses revealed a significant positive association of higher CONUT scores with DR risk in older female patients with diabetes, alcohol use, smoking history, hypertension, and hyperlipidemia.

**Conclusion:**

There is a sex difference in the link between higher CONUT scores and the prevalence of DR. Specifically, these findings highlight the importance of personalized nutritional intervention in women at high risk for DR.

## 1 Introduction

Type 2 diabetes mellitus (T2DM) is a chronic metabolic disorder characterized by elevated blood glucose levels (1). T2DM is becoming increasingly prevalent and has become a major public health challenge worldwide (2). Long-term impairment caused by hyperglycemia predisposes T2DM to various serious complications, including cardiovascular disease, nephropathy, neuropathy, and retinopathy (3, 4). Diabetic retinopathy (DR) is the most common and serious microvascular complication of T2DM and a leading cause of vision loss in adults (5). It is estimated that approximately one-third of T2DM patients worldwide develop DR (6, 7). Risk factors for DR include hyperglycemia, hypertension, and the duration of diabetes (8). The incidence of DR increases with the duration of diabetes and poor glycemic control. Notably, sex differences may contribute to the incidence, duration, and complications of diabetes. Studies have demonstrated that male T2DM patients typically exhibit a higher risk of cardiovascular disease, while female patients are more likely to be affected by vascular complications such as DR (9). This difference may be attributed to sex hormone levels, lipid metabolism, and sex-specific immune responses. Additionally, decreased estrogen levels in women after menopause may further increase their risk of developing diabetic complications (10). Although glycemic control and blood pressure management have been widely accepted as important means of DR prevention and treatment, the specific presentations and clinical significance of sex differences in DR still warrant further investigation.

There has been a growing interest in studying the impact of nutritional status on DR (11). Good nutritional status not only affects overall health but also plays an important role in the management of diabetes and its complications. Single nutritional measures, such as serum albumin (ALB), have been shown to play an important role in predicting nutritional status and outcomes in patients with chronic diseases (e.g., diabetes, hypertension, and COPD) (12, 13). Moreover, additional complex measures have been developed and validated for assessing nutritional status (14, 15). Özdemir et al. explored the impact of malnutrition biomarkers and cardiometabolic control on diabetic retinopathy, further validating the utility of nutritional assessment tools in predicting diabetic complications (16). Therefore, the application of effective nutritional assessment tools is crucial for monitoring and improving the nutritional status of DR patients. The Controlling Nutritional Status (CONUT) score is a nutritional screening tool first proposed in 2005 (17). Unlike many nutritional assessment tools that rely on a single or limited number of parameters, the CONUT score incorporates three key nutritional and immune markers, namely serum albumin (ALb), total lymphocyte count (TL), and total cholesterol (TC), to provide a more comprehensive evaluation of both nutritional status and immune function. This multidimensional approach allows for effective identification of malnutrition risk, assessment of overall nutritional condition, and prediction of potential complications, making the CONUT score a valuable tool for guiding clinical decision-making. Although studies have revealed an association between nutritional status and DR progression, how to assess DR using systematic, quantitative nutritional assessment tools such as CONUT and determine its sex differences remains unclear (18, 19). Therefore, further investigation into the application of the CONUT score in DR patients, particularly its performance across sexes, is anticipated to offer insights for developing more accurate and sex-specific nutritional intervention strategies.

No study thus far has examined the relationship between the CONUT score and DR and their sex differences. Therefore, this study examined the association of CONUT score with DR in male and female adults who participated in the National Health and Nutrition Examination Survey (NHANES) from 2003 to 2018.

## 2 Materials and methods

### 2.1 Data source and participants

**Figure 1:**
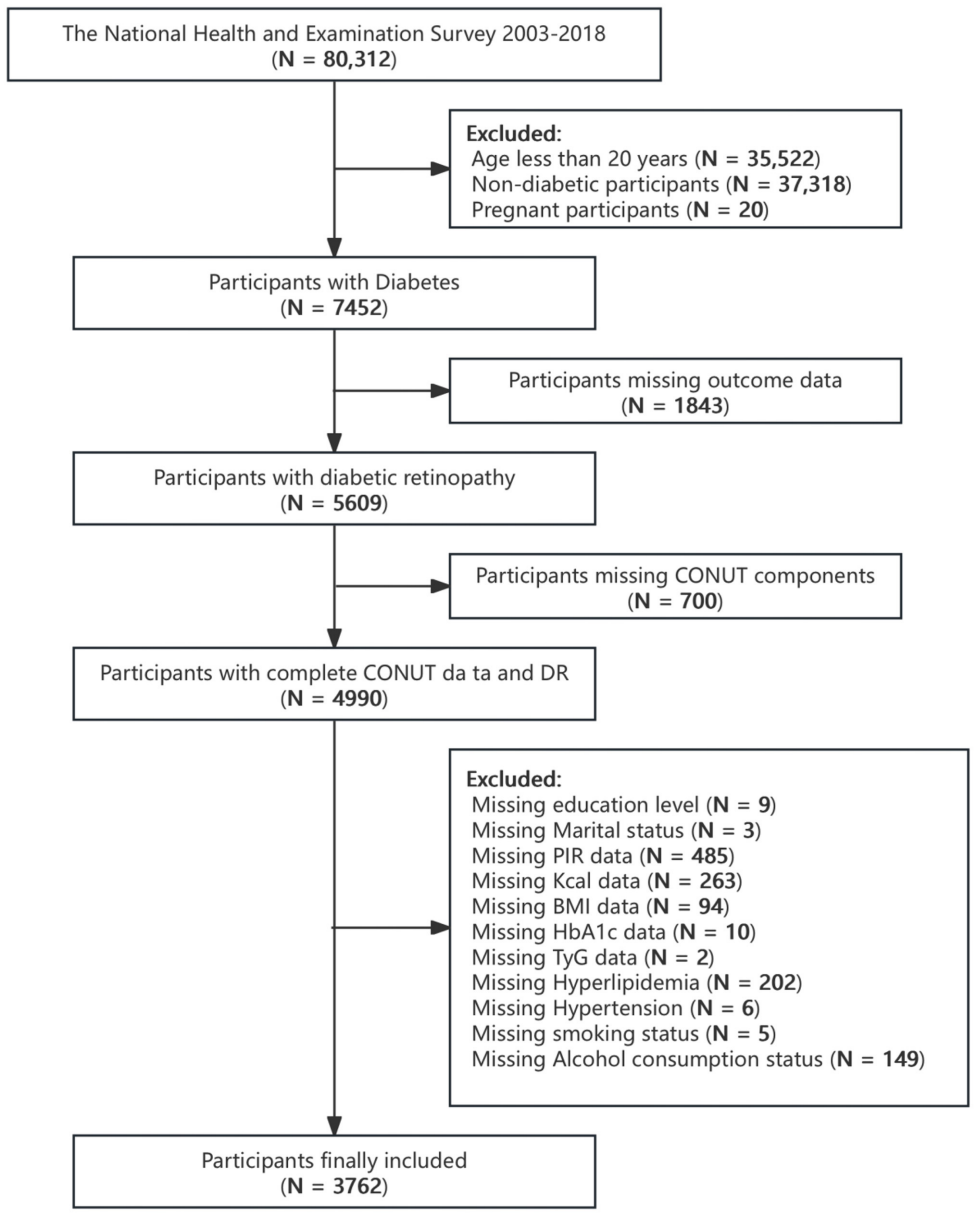
Flowchart of the selection strategy.

The NHANES, conducted by the Centers for Disease Control and Prevention (CDC), is a nationally representative survey designed to assess the health and nutritional status of adults and children in the United States using a complex multi-stage probability sampling design. Demographic, dietary, examination, laboratory, and questionnaire data are collected and published by the National Center for Health Statistics (NCHS) of CDC every 2 years. In this cross-sectional study, relevant data from individuals aged 20 or older were extracted from eight NHANES cycles from 2003 to 2018 for analysis. Diabetes was defined as physician-reported diagnosis, fasting blood glucose (FBG) ≥7 mmol/L, glycosylated hemoglobin (HbA1c) ≥6.5%, use of antidiabetic medications, or use of insulin (20). Individuals who failed to meet the definition of diabetes, had missing measures for diabetes diagnosis, were pregnant, or had missing outcomes, CONUT components or covariate data were excluded. A final total of 3,762 participants were included in this study (Figure 1).

### 2.2 Assessment of diabetic retinopathy

For the diabetes section of the NHANES, the questionnaire was conducted at home by a trained interviewer using the computer-assisted personal interview (CAPI) system. The CAPI system has built-in consistency checks to minimize data entry errors and offers online help screens to help interviewers define key terms in the questionnaire. To determine the diagnosis of diabetes, the subject was asked to complete a diabetes questionnaire that included a question: “Has a doctor ever told [you/he/she] that diabetes affected [your/his/her] eyes, or that [you/he/she] had retinopathy? A response of “Yes” was considered having DR.

### 2.3 Assessment of CONUT

The CONUT score was developed by Ignacio de Ulíbarri et al. (17) and is composed of three parameters, namely ALB, TC and TL. The optimal CONUT score cutoff was determined to be 2.5 using the receiver operating characteristic curve (ROC) (21, 22). Therefore, participants were grouped according to CONUT < 2.5 and CONUT ≥2.5. Details of CONUT are shown in Supplementary Table 1.

### 2.4 Assessment of covariates

The covariates included in this study were age (extracted from demographic records of family interviews), race (non-Hispanic White, non-Hispanic Black, Mexican American, or other), smoking (never smoked or current smoker), alcohol use (in the past 1 year), marital status (married, widowed/divorced/separated, or single), body mass index (BMI; kg/m^2^), hypertension and hyperlipidemia (self-reported, yes or no), education level (below high school, high school, above high school), poverty to income ratio (PIR; poverty measured by total household income/poverty line), hemoglobin, energy, carbohydrates, total fat, vitamin B6, vitamin B12, and glycated hemoglobin.

### 2.5 Statistical analysis

All analyses were conducted in accordance with NHANES dataset guidelines, using primary sampling units, pseudo-variance, and masked variance in sampling weights to adjust for the multi-stage sampling design and provide representative estimates. Since the covariates selected encompassed the first day of dietary intake data, which was of higher quality and more representative, the Day 1 dietary sampling weight (1/8 ^*^ WTDRD1) was used for the analysis. The baseline characteristics of the participants were grouped according to the presence or absence of DR. Continuous variables are presented as median (P25, P75). Categorical variables are expressed as the number of participants (percentages) and compared using the Pearson's chi-square test. Continuous variables were not normally distributed (Shapiro-Wilk test) and were hence compared using the Mann–Whitney U-test.

The link between CONUT score and DR risk in the overall population was analyzed using multivariate logistic regression and stratified by sex. The results are expressed as odds ratio (OR) and 95% confidence interval (CI).

CONUT scores were first analyzed as a continuous variable and then classified into two categories (< 2.5 and ≥2.5). The model was constructed by stepwise adjustment of covariates: model 1: no adjustment for covariates; model 2: adjustments for sex, age, race, education, marital status, and PIR; model 3: additional adjustments for BMI, hemoglobin, energy, carbohydrates, total fat, vitamin B6, vitamin B12, glycosylated hemoglobin, hypertension, hyperlipidemia, smoking, and alcohol use based on model 2. Additionally, restricted cubic spline (RCS) models were fitted separately for the overall population, as well as for male and female subpopulations, to evaluate the non-linear relationship between CONUT score and DR. Subsequently, subgroup analyses based on sex were performed to determine the modifying effects of key demographic and clinical variables on the relationship between CONUT and outcomes. All covariates (except those used for stratification) were adjusted in the model. These analyses were conducted based on age (< 60 or ≥60), race (Mexican American/Non-Hispanic White/Non-Hispanic Black/Other), alcohol use (yes/no), education level (below high school/high school/above high school), marital status (married, widowed/divorced/separated, or single), smoking (never smoked/former smoker/current smoker), hypertension (yes/no), and hyperlipidemia (yes/no). The interaction between CONUT score and these variables was assessed by incorporating the interaction terms in the multivariate logistic regression model. Specifically, a multiplicative interaction term (CONUT × covariate) was incorporated to assess whether the association between CONUT score and DR interacted with other variables. The significance of interaction was tested using the analysis of variance. Propensity score matching (PSM) was employed to minimize potential selection bias and confounding factors. Propensity scores were estimated using a logistic regression model incorporating relevant covariates, followed by 1:1 nearest-neighbor matching of patients. Covariate balance between matched groups was evaluated using standardized mean differences (SMDs), with an SMD < 0.1 indicating adequate balance. A weighted logistic regression model was then used to analyze the matched data and assess the association between CONUT scores and DR between genders, improving the validity and robustness of the study results. All statistical analyses were performed using R 4.3.0. A *P* < 0.05 indicates statistical significance.

## 3 Results

### 3.1 Baseline characteristics of the population

The individual baseline characteristics of participants with and without DR are summarized in Table 1. This study involved 3,762 DR patients over the age of 20. The median age was 61 years, with 52% of participants being male, 48% female, and 64% non-Hispanic White. DR is usually associated with decreased poverty and higher ALB, white blood cells, red blood cells, lymphocytes, and glycated hemoglobin levels.

**Table 1:** Baseline information from the DR and non-DR groups.

**Characteristic**	***N* ^a^**	**Overall, *N =* 3,762^b^**	**Non-diabetic retinopathy, *N =* 2,991^b^**	**Diabetic retinopathy, *N =* 771^b^**	***p*-value^c^**
Gender	3,762				0.324
Male		2,001 (52%)	1,573 (51%)	428 (54%)	
Female		1,761 (48%)	1,418 (49%)	343 (46%)	
Age	3,762	61 (52,70)	62 (52,70)	60 (52,70)	0.834
Race	3,762				0.451
Mexican American		679 (8.4%)	546 (8.5%)	133 (8.0%)	
Non-Hispanic White		1,459 (64%)	1,181 (65%)	278 (64%)	
Non-Hispanic Black		955 (14%)	750 (14%)	205 (16%)	
Other Race		669 (13%)	514 (12.5%)	155 (12%)	
Education	3,762				0.06
< High school diploma		1,244 (23%)	970 (22%)	274 (27%)	
High school diploma/equivalent		876 (25%)	697 (25%)	179 (26%)	
>High school diploma		1,642 (52%)	1,324 (53%)	318 (47%)	
Marital	3,762				0.459
Married/cohabitation		2,298 (65%)	1,841 (65%)	457 (63%)	
Widow/divorce/separation		1,173 (28%)	917 (27%)	256 (30%)	
Unmarried		291 (7.6%)	233 (7.8%)	58 (6.8%)	
PIR	3,762	2.50 (1.31, 4.51)	2.58 (1.34, 4.52)	2.10 (1.12, 4.37)	0.015
BMI	3,762	32 (28, 37)	32 (28, 37)	32 (28, 38)	0.878
Albumin	3,762	4.10 (3.90, 4.40)	4.20 (4.00, 4.40)	4.10 (3.90, 4.30)	< 0.001
TyG	3,762	163 (108, 239)	165 (109, 239)	156 (107, 242)	0.721
Leukocyte	3,762	7.50 (6.30, 9.00)	7.57 (6.40, 9.10)	7.30 (5.90, 8.80)	0.016
Lymphocyte	3,762	2.00 (1.60, 2.60)	2.10 (1.60, 2.60)	2.00 (1.50, 2.60)	0.046
Erythrocyte	3,762	4.66 (4.32, 4.99)	4.68 (4.33, 5.01)	4.57 (4.25, 4.92)	0.001
Hemoglobin	3,762	14.10 (13.00, 15.00)	14.10 (13.10, 15.00)	13.80 (12.60, 14.80)	< 0.001
RDW	3,762	13.40 (12.80, 14.20)	13.40 (12.80, 14.20)	13.50 (12.70, 14.20)	0.777
Theombocyte	3,762	233 (192, 283)	234 (194, 286)	229 (189, 273)	0.054
HbA1c	3,762	6.80 (6.20, 7.90)	6.70 (6.10, 7.70)	7.30 (6.50, 8.56)	< 0.001
HDL-C	3,762	45 (38, 54)	45 (38, 54)	46 (37, 55)	0.533
Total cholesterol	3,762	176 (150, 208)	177 (151, 208)	174 (146, 211)	0.651
Kcal	3,762	1,765 (1,314, 2,339)	1,776 (1,320, 2,333)	1,675 (1,262, 2,340)	0.291
Carbohydrate	3,762	202 (150, 270)	203 (150, 269)	199 (145, 275)	0.632
Total fat	3,762	69 (47, 102)	70 (48, 102)	65 (44, 100)	0.197
Vitamin B6	3,762	1.65 (1.11, 2.30)	1.66 (1.11, 2.29)	1.63 (1.07, 2.31)	0.831
Vitamin B12	3,762	3.6 (2.1, 5.8)	3.7 (2.1, 5.9)	3.5 (2.1, 5.4)	0.326
Hypertension	3,762	2,657 (70%)	2,076 (69%)	581 (74%)	0.06
Hyperlipidemia	3,762	2,409 (66%)	1,892 (65%)	517 (68%)	0.254
Drink	3,762	2,536 (71%)	2,022 (71%)	514 (68%)	0.24
Smoking	3,762				0.245
Never		1,826 (48%)	1,455 (48%)	371 (48%)	
Former		1,359 (36%)	1,061 (36%)	298 (39%)	
Current		577 (16%)	475 (16%)	102 (13%)	
COUNT	3,762	1 (0,2)	1 (0,2)	1 (0,2)	0.062

Continuous variables are presented as weighted means (SE). Categorical variables are expressed as counts (weighted percentages).

^a^*N* not Missing (unweighted).

^b^*n* (unweighted) (%); Median (25%, 75%).

^c^Chi-squared test with Rao and Scott's second-order correction; Wilcoxon rank-sum test for complex survey samples.

PIR, Poverty-income ratio; BMI, Body mass index; TyG, Triglyceride glucose index; RDW, Red cell distribution width; HbA1c, Glycosylated hemoglobin; HDL-C, High density lipoprotein cholesterol.

### 3.2 Overall association between CONUT score and DR

Multivariate logistic regression analysis revealed a significant positive association between CONUT score and DR. This association was significant in both model 1 (OR = 1.12, 95% CI: 1.03–1.23) and model 2 (OR = 1.13, 95% CI: 1.02–1.25), but not in model 3 (OR = 1.09, 95% CI: 0.97–1.23). After converting CONUT score from a continuous variable to a categorical variable, further analysis showed that there was a significant positive association between CONUT score and DR risk in model 1 (OR = 1.61, 95% CI: 1.18–2.20), model 2 (OR = 1.60, 95% CI: 1.15, 2.22), and model 3 (OR = 1.46, 95% CI: 1.01, 2.09) when CONUT score was ≥2.5 but not < 2.5 (Table 2).

**Table 2:** The association between CONUT score and diabetic retinopathy (DR).

**Variable**	**Characteristic**	**Model 1 OR (95%CI)**	***P*-value**	**Model 2 OR (95%CI)**	***P*-value**	**Model 3 OR (95%CI)**	***P*-value**
Overall	CONUT	1.12 (1.03, 1.23)	*P =* 0.012	1.13 (1.02, 1.25)	*P =* 0.023	1.09 (0.97, 1.23)	*P =* 0.13
	CONUT Group						
	< 2.5	–		–		–	
	>2.5	1.61 (1.18, 2.20)	*P =* 0.003	1.60 (1.15, 2.22)	*P =* 0.005	1.46 (1.01, 2.09)	*P =* 0.043
Female	CONUT	1.25 (1.09, 1.45)	*P =* 0.002	1.24 (1.07, 1.43)	*P =* 0.005	1.25 (1.06, 1.48)	*P =* 0.009
	CONUT Group						
	< 2.5	–		–		–	
	>2.5	1.94 (1.19, 3.14)	*P =* 0.008	1.83 (1.14, 2.94)	*P =* 0.013	1.88 (1.13, 3.15)	*P =* 0.016
Male	CONUT	1.03 (0.91, 1.17)	*P =* 0.7	1.07 (0.93, 1.22)	*P =* 0.4	1.02 (0.88, 1.17)	*P =* 0.8
	CONUT Group						
	< 2.5	–		–		–	
	>2.5	1.42 (0.95, 2.14)	*P =* 0.088	1.54 (1.01, 2.35)	*P =* 0.046	1.38 (0.86, 2.20)	*P =* 0.2

Data are presented as weighted odds ratios (OR) with 95% confidence intervals (CI). Model 1 is the crude model. Model 2 is adjusted for gender, age, race, education, marital status, and PIR. Model 3 is further adjusted for BMI, hemoglobin, energy intake, carbohydrates, total fat, vitamin B6, vitamin B12, glycated hemoglobin, hypertension, hyperlipidemia, smoking, and alcohol consumption.

### 3.3 Association between CONUT score and DR among males and females

**Figure 2:**
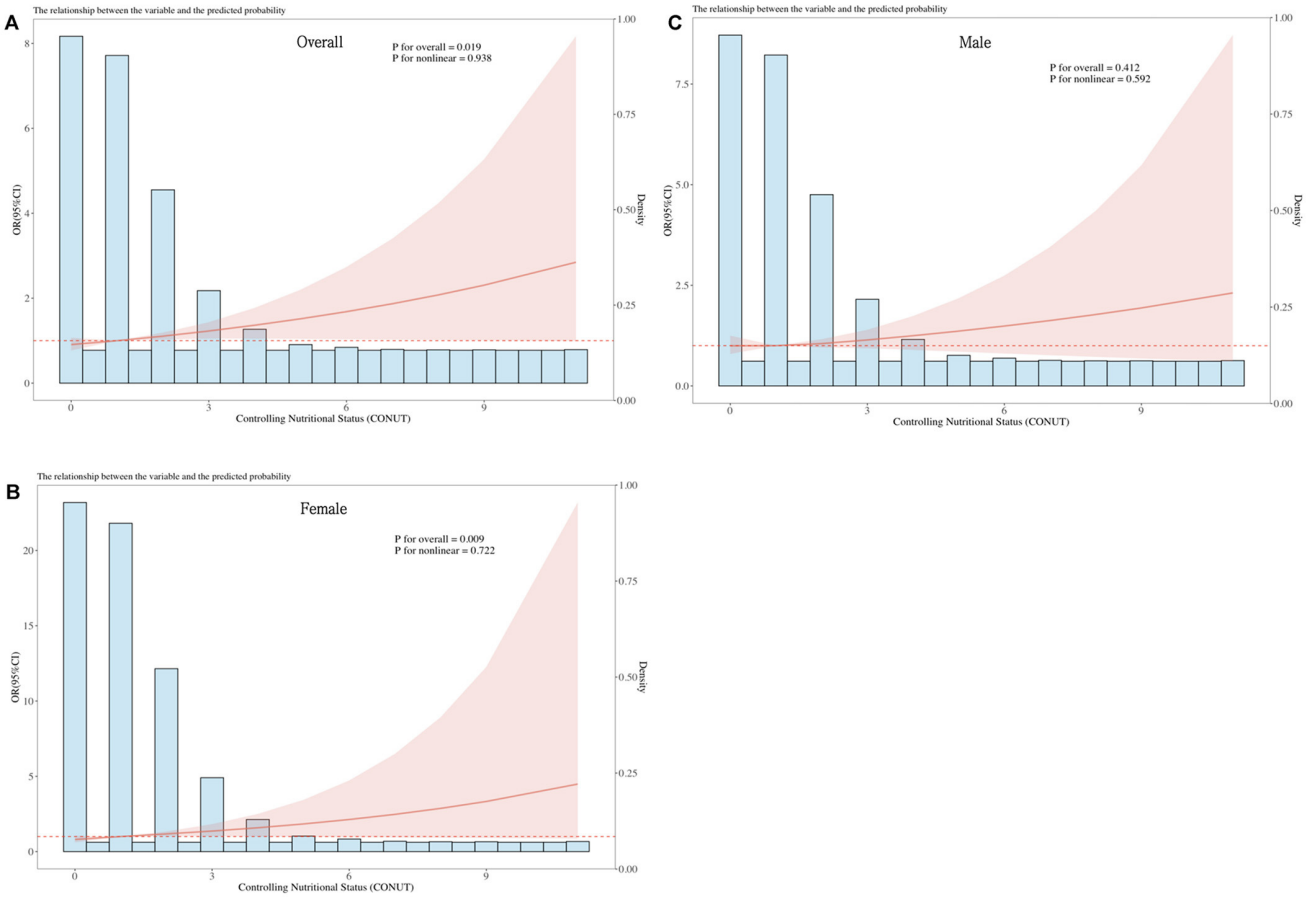
Dose-response relationship between CONUT and overall DR risk and sex-specific DR risk. The model is adjusted for gender, age, race, education, marital status, PIR, BMI, hemoglobin, energy intake, carbohydrates, total fat, vitamin B6, vitamin B12, glycated hemoglobin, hypertension, hyperlipidemia, smoking, and alcohol consumption. The central estimates are represented by the solid red line, the red shaded area represents the 95% confidence intervals, and the frequency density is depicted by the blue bar graph. **(A)** Dose-response relationship between CONUT and the total population with DR. **(B)** Dose-response relationship between CONUT and female patients with DR. **(C)** Dose-response relationship between CONUT and male patients with DR.

We identified sex differences in the relationship between CONUT score and DR (Table 2). As we further adjusted for covariates, a higher CONUT score was more detrimental for women than for men. In particular, model 3 demonstrated that CONUT score was significantly positively correlated with DR risk in females **(OR** **=** **1.25, 95% CI: 1.06–1.48)**. In addition, DR risk was 88% higher in the high CONUT score group (≥2.5) compared to the low CONUT score group (< 2.5) **[OR** **=** **1.88 (95% CI: 1.13–3.15)]**. However, no significant association was observed in male participants. Additionally, multivariate-adjusted RCS plots revealed positive linear trends in CONUT scores for all participants (*P*-Non-linear: 0.983) and female participants (*P*-Non-linear: 0.722) (Figure 2).

### 3.4 Subgroup analyses

**Figure 3:**
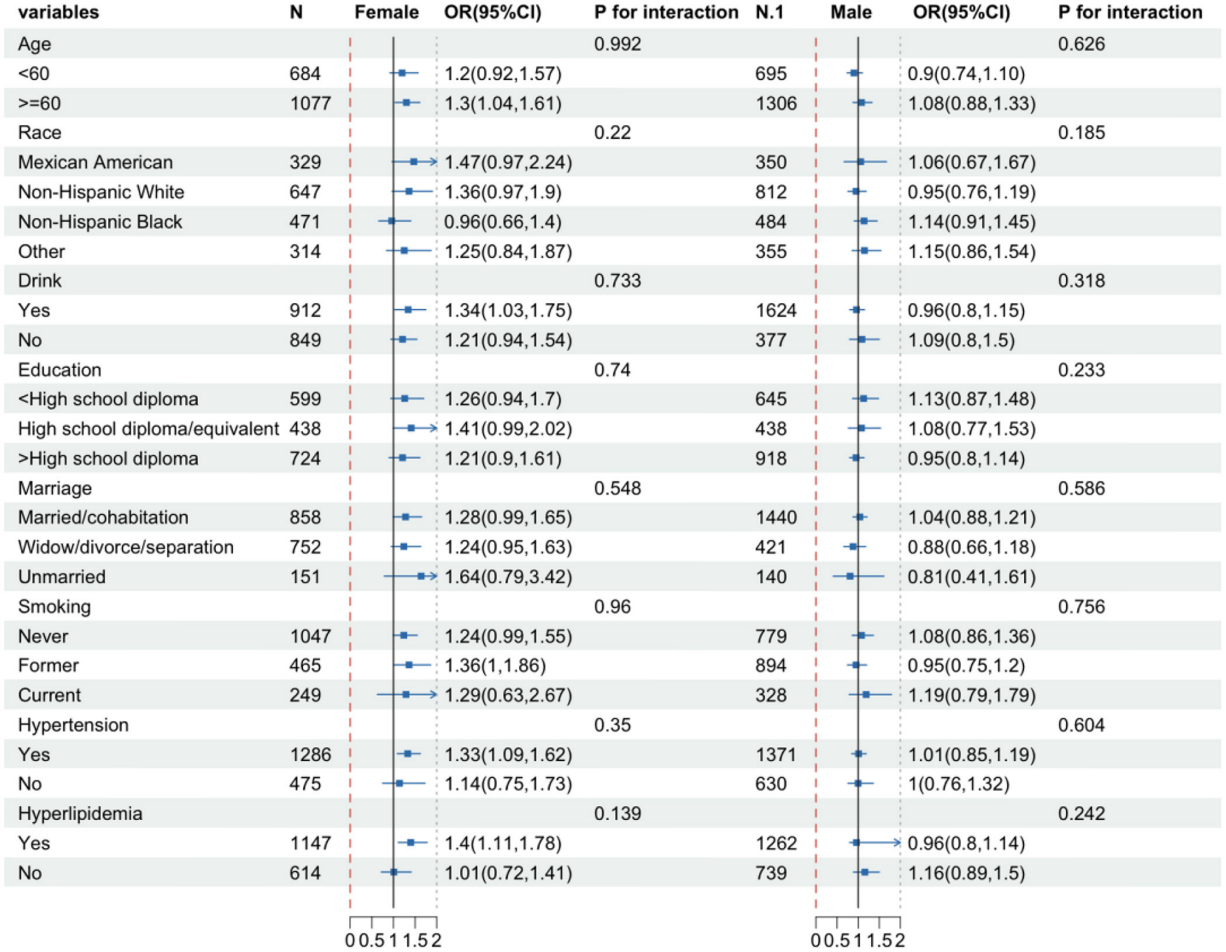
Subgroup analysis of female and male groups. The model is adjusted for gender, age, race, education, marital status, PIR, BMI, hemoglobin, energy intake, carbohydrates, total fat, vitamin B6, vitamin B12, glycated hemoglobin, hypertension, hyperlipidemia, smoking, and alcohol consumption.

To determine whether there was a substantial association between CONUT scores and DR in specific subgroups, we performed subgroup analyses of CONUT scores separately for females and males. Participants were subgrouped based on sex, race, education level, marital status, smoking, alcohol use, hypertension, and hyperlipidemia, and the analyses were conducted using logistic regression. All covariates were adjusted in the model except for those used for stratification. As shown in Figure 3, there was a significant positive correlation of CONUT score with DR in females who were aged ≥60 years **(OR** **=** **1.3, 95% CI: 1.04, 1.61)**, drinking **(OR** **=** **1.34, 95% CI: 1.03, 1.75)**, former smokers **(OR** **=** **1.36, 95% CI: 1, 1.86)**, and suffering from hypertension **(OR** **=** **1.33, 95% CI: 1.09, 1.62)** and hyperlipidemia **(OR** **=** **1.4, 95% Cl: 1.11, 1.78)**. However, no significant association was observed in male participants. The *P*-value of the interaction term indicated no significant interaction between the CONUT score and each variable in males and females (*P*-interaction >0.05).

### 3.5 Sensitivity analysis

To account for confounding factors, PSM and multivariable logistic regression analysis were performed, adjusting for various potential confounders in the original (non-matched) data. The results were consistent with the primary estimates reported. In the matched cohort, the fully adjusted model revealed a significant positive association between CONUT scores and the risk of DR among female participants (**OR** **=** **1.35, 95% CI: 1.12–1.63**; Supplementary Table 2). When CONUT scores were categorized, participants with higher CONUT scores (≥2.5) had a 147% increased risk of DR compared to those with lower CONUT scores (< 2.5) **[OR** **=** **2.47 (95% CI: 1.33–4.59)]**. However, no significant association between CONUT scores and DR was observed among male participants.

## 4 Discussion

This study analyzed the association between the Controlling Nutritional Status (CONUT) score and the prevalence of diabetic retinopathy (DR) in the general population, along with sex-based differences. Initial analyses (Models 1 and 2) showed a statistically significant positive correlation between the CONUT score and DR risk in the overall population. However, this association was not statistically significant in the fully adjusted model. Further analysis by sex revealed a significant association between higher CONUT scores and increased DR risk in females. Furthermore, subgroup analyses showed that among females who were older and had a history of alcohol consumption or smoking, or had hypertension or hyperlipidemia, a significant positive correlation between the CONUT score and DR was observed.

Previous studies have identified a link between nutritional risk and diabetes (23). According to previous reports, malnourished patients with type 2 diabetes mellitus (T2DM) exhibit elevated levels of glycated hemoglobin (HbA1c), random blood glucose (RBG), insulin, and glucagon, likely due to reduced insulin sensitivity and increased insulin resistance (24). Caputo et al. (25) demonstrated that, under malnutrition, the GH/IGF-1 axis prioritizes protein reserve preservation by enhancing lipolysis and suppressing carbohydrate oxidation. This axis simultaneously regulates metabolic balance through negative feedback, inhibiting insulin signaling and promoting fatty acid oxidation, which impacts glucose homeostasis and energy metabolism. Additionally, malnutrition not only affects metabolic and endocrine functions in diabetic patients, but also significantly alters the expression of immune-related cytokines such as IL-2, IL-8, and IL-21 (26). The reduction in these cytokines weakens immune defense mechanisms in diabetic patients, increasing their susceptibility to infections. Moreover, malnutrition exacerbates the chronic inflammatory state in diabetic patients by influencing the expression of inflammatory factors such as TNF-α and IL-6 (27). Therefore, malnutrition involves a complex, interconnected cascade of metabolic dysregulation, endocrine abnormalities, and immune dysfunction. These mechanisms can further deteriorate systemic metabolic function and may also compromise the overall health status of diabetic patients by affecting nutrient absorption and utilization. The high prevalence of diabetes-related complications and comorbidities may further impair nutritional status (28, 29).

In diabetic patients with diabetic retinopathy (DR), several factors can impair retinal blood supply and cellular function, leading to reduced nutrient utilization. These factors include oxidative stress, chronic inflammation caused by prolonged hyperglycemia, medication use, nutrient deficiencies, and diabetic nephropathy (19, 30–32). These factors not only directly damage retinal cell health but may also worsen malnutrition by affecting systemic metabolism, nutrient absorption, and distribution. Moreover, studies exploring the relationship between nutritional status and DR have primarily focused on the correlation between malnutrition and DR. For example, studies suggest that vitamin D deficiency is linked to an increased risk of DR, and low vitamin D levels may accelerate retinopathy progression (33, 34). However, studies examining the impact of nutritional status on DR, considering sex differences, remain limited. Nevertheless, some studies have investigated sex-related differences in DR. A large pooled analysis showed similar prevalence rates of diabetic retinopathy in both males and females (35). In contrast, other studies have reported higher prevalence and severity of retinopathy, as well as faster progression, in males (36–39). However, some studies have reported the opposite trend (40). For example, a retrospective longitudinal study in Japan found a significantly higher prevalence of diabetic retinopathy (DR) in females, with female sex identified as an independent risk factor for DR development (41).

Building on these findings, this study introduces the Controlling Nutritional Status (CONUT) score, a multidimensional nutritional assessment tool, to offer a comprehensive and timely evaluation of nutritional status in patients with diabetic retinopathy (DR). The CONUT score, based on three objective biomarkers (17), has gained increasing recognition in recent years for its use in studying diabetes and its complications. By assessing serum albumin, total cholesterol, and lymphocyte count—three key indicators—the CONUT score provides a comprehensive reflection of a patient's nutritional status. Specifically, serum albumin reflects protein reserves, total cholesterol evaluates energy consumption, and lymphocyte count indicates the impact of nutritional status on immune function (17). Previous studies have demonstrated the clinical value of the CONUT score in predicting carotid atherosclerosis, diabetic foot ulcers, renal insufficiency, and mortality risk in diabetic patients (42–45). Although the importance of the CONUT score in various diabetic complications is widely acknowledged, research on its association with diabetic retinopathy (DR) remains limited. Wei et al. (23) assessed the nutritional status of 612 DR patients using the Global Leadership Initiative on Malnutrition (GLIM) criteria, CONUT, Nutritional Risk Index (NRI), and Prognostic Nutritional Index (PNI) to investigate the relationship between malnutrition and DR. Their study showed that higher CONUT scores were associated with a higher incidence of DR, consistent with the results of our classification model. However, that study did not observe sex-related differences in the association between malnutrition and DR. In contrast, our study uses a more representative data source and incorporates a wider range of socioeconomic and more detailed nutritional factors in the covariate adjustment. Additionally, we adjusted for key diabetes control indicators, such as glycated hemoglobin. Our study extends the investigation of the impact of the CONUT score on DR by revealing sex differences in how nutritional status affects DR. We found that female had a higher risk of DR than male, with a more pronounced relationship between CONUT score and DR risk in female, while no significant association was found in male.

When investigating sex differences in the association between the CONUT score and diabetic retinopathy (DR), estrogen may play a critical role. Female individuals experience significant hormonal shifts throughout their lives, including during menstrual cycles, pregnancy, postpartum periods, and menopause (46–48). During these periods, estrogen and progesterone levels fluctuate significantly. Particularly after menopause, the marked decline in estrogen levels can increase susceptibility to chronic low-grade inflammation and metabolic abnormalities, which may place female individuals at higher risk for DR (49, 50). Estrogen is crucial for maintaining vascular endothelial function, regulating cell apoptosis, and promoting antioxidant defenses. Estrogen deficiency may reduce microvascular stability and enhance inflammatory responses, exacerbating retinal microcirculatory abnormalities (51). Notably, fluctuations in estrogen levels may indirectly affect the CONUT score by influencing nutritional status. The CONUT score, a comprehensive indicator of a patient's nutritional status, is influenced by the body's endocrine and metabolic state (52).

It is important to note that although the CONUT score does not directly measure hormone levels or metabolic pathways, its components may be potentially linked to estrogen-related mechanisms. ALB, a key component of the CONUT score, not only serves as a marker of nutritional status but also plays a critical role in vascular function and inflammation regulation (53). Estrogen deficiency can elevate systemic inflammatory markers (e.g., CRP, IL-6) and increase oxidized low-density lipoprotein (ox-LDL) levels (54–56). This chronic inflammatory state suppresses ALB synthesis, leading to reduced serum ALB levels that may compromise endothelial function and microcirculatory stability. Serum TC levels are also modulated by estrogen. A study in Pakistani women reported a significant postmenopausal decrease in HDL-C levels (*P* < 0.001), along with substantial increases in LDL-C and very-low-density lipoprotein (VLDL) levels (57). These changes disrupt lipid homeostasis, promoting vascular lipid deposition and exacerbating vascular damage. TL is the third component of the CONUT score and a marker of immune function. Estrogen regulates lymphocyte count and function through various mechanisms and plays a key role in maintaining microvascular endothelial health. For example, estrogen modulates T cell subsets (e.g., CD4^+^ T cells and regulatory T cells), inhibits inflammation-induced lymphocyte migration, and preserves microvascular blood flow by protecting endothelial function (58, 59). Additionally, estrogen modulates the expression of adhesion molecules to govern the interaction between lymphocytes and endothelial cells. The integrity of the microvascular endothelium (e.g., blood-brain barrier) is closely linked to immune cell infiltration (60, 61). Collectively, these mechanisms suggest that estrogen-driven changes in lymphocyte dynamics may serve as a biomarker for immune-microvascular interactions. Moreover, female individuals experience changes in fat distribution and metabolism at different life stages, especially after menopause, when abdominal fat accumulation often becomes more pronounced. A Latin American study showed that the association between abdominal obesity and diabetes was more pronounced in female individuals, and that even in individuals with normal weight, especially female individuals, abdominal fat accumulation significantly increased the risk of diabetes (62). This change in fat distribution may increase the risk of diabetes-related retinopathy. These sex-specific physiological characteristics may make female microvascular systems more susceptible to the effects of malnutrition.

This study has several highlights. First, we used nationally representative data from NHANES, a large and diverse database that provides strong evidence for assessing sex differences. Second, this is the first study to examine the sex differences in the relationship between CONUT score and DR, addressing a current research gap. However, certain limitations should be considered. First, the cross-sectional nature of this study prevented the assessment of causality. Future studies should consider employing approaches such as Mendelian randomization to explore its causal relationship. Second, the diagnosis of DR relied on self-reported data, which may introduce potential bias. Third, data limitations prevented further exploration of the long-term impact of nutritional interventions on DR progression, warranting future longitudinal studies. Last, since this study was conducted using representative samples from the US, the findings may have limited generalizability to other populations. Therefore, recruiting participants from different cultural or geographical backgrounds is necessary for subsequent multicenter clinical studies.

## 5 Conclusions

High CONUT scores are significantly positively correlated with DR prevalence in women, highlighting the key role of nutritional status assessment in managing diabetic complications. Our findings offer valuable insights into the improvement of personalized nutritional intervention strategies for women with diabetes and open avenues for future studies on nutritional interventions targeting the prevention and treatment of DR.

## Data Availability

The raw data supporting the conclusions of this article will be made available by the authors, without undue reservation.
