# Supplementary material for: Sex differences in the impact of controlling nutritional status score on diabetic retinopathy: findings of 2003–2018 National Health and Nutrition Examination Survey
Source: Front Nutr. 2025 May 16;12:1597521. doi: 10.3389/fnut.2025.1597521 (PMC12135625; doi:10.3389/fnut.2025.1597521)
Supplement: Supplementary file 1 [file Table_1.docx]

**Supplementary Table1.** Calculation of CONUT Score

| Parameters | CONUT | | | |
| --- | --- | --- | --- | --- |
|  | Normal | Light | Moderate | Severe |
| Serum albumin (g/dL) | 3.5-4.5 | 3.0-3.49 | 2.5-2.9 | <2.5 |
| Alb score | 1 | 2 | 4 | 6 |
| Total lymphocyte (count/mm^3^) | ≥1600 | 1200-1599 | 800-1199 | <800 |
| TLC score | 0 | 1 | 2 | 3 |
| Total cholesterol (mg/dL) | >180 | 140-180 | 100-139 | <100 |
| T-cho score | 0 | 1 | 2 | 3 |

CONUT is calculated as the sum of the Alb score, TLC score, and T-cho score

Alb albumin, TLC total lymphocytes, T-cho total cholesterol
