# Supplementary material for: Sex differences in the impact of controlling nutritional status score on diabetic retinopathy: findings of 2003–2018 National Health and Nutrition Examination Survey
Source: Front Nutr. 2025 May 16;12:1597521. doi: 10.3389/fnut.2025.1597521 (PMC12135625; doi:10.3389/fnut.2025.1597521)
Supplement: Supplementary file 2 [file Table_2.docx]

**Supplementary Table 2.** The Association Between CONUT Score and Diabetic Retinopathy (DR) After Propensity Score Matching

| Variable | Characteristic | Model1 OR(95%CI) | P-value | Model2 OR(95%CI) | P-value | Model3 OR(95%CI) | P-value |
| --- | --- | --- | --- | --- | --- | --- | --- |
| Female | CONUT | 1.26(1.03,1.54) | 0.025 | 1.23(1.00,1.51) | 0.049 | 1.35(1.12,1.63) | 0.002 |
|  | CONUT Group |  |  |  |  |  |  |
|  | <2.5 | —— |  | —— |  | —— |  |
|  | >=2.5 | 2.01(1.08,3.73) | 0.027 | 1.91(1.01,3.61) | 0.047 | 2.47(1.33,4.59) | 0.005 |
| Male | CONUT |  |  |  |  |  |  |
|  | CONUT Group | 0.96(0.82,1.11) | 0.5 | 1.00(0.84,1.18) | 0.9 | 1.00(0.85,1.17) | 0.9 |
|  | <2.5 | —— |  | —— |  | —— |  |
|  | >=2.5 | 1.14(0.67,1.94) | 0.6 | 1.37(0.76,2.47) | 0.3 | 1.32(0.76,2.29) | 0.3 |

**Notes:** Data are presented as weighted odds ratios (OR) with 95% confidence intervals (CI). Model 1 is the crude model. Model 2 is adjusted for gender, age, race, education, marital status, and PIR. Model 3 is further adjusted for BMI, hemoglobin, energy intake, carbohydrates, total fat, vitamin B6, vitamin B12, glycated hemoglobin, hypertension, hyperlipidemia, smoking, and alcohol consumption.
